# Transdiagnostic factors in symptoms of depression and post-traumatic stress: a systematic review

**DOI:** 10.1007/s12144-023-04792-x

**Published:** 2023-05-29

**Authors:** Alejandrina Hernández-Posadas, Miriam J. J. Lommen, Anabel de la Rosa Gómez, Theo K. Bouman, Juan Manuel Mancilla-Díaz, Adriana del Palacio González

**Affiliations:** 1 Faculty of Behavioural and Social Sciences, University of Groningen, Groningen, Netherlands; 2 Faculty of Higher Studies Iztacala, National Autonomous University of Mexico, Av. De los Barrios Núm. 1, Los Reyes Iztacala, Torre de Tutorías, 2do. Piso, Cubículo 22, Edo. de Mexico 54090 Tlalnepantla, Mexico; 3 Center for Alcohol and Drug Research, Aarhus University, Aarhus, Denmark

**Keywords:** Intolerance of uncertainty, Emotional dysregulation, Rumination, Depression, Post-traumatic stress, Systematic review

## Abstract

The current systematic review sought to identify quantitative empirical studies that focused on the transdiagnostic factors of intolerance of uncertainty, emotional dysregulation and rumination, and their relation with depression and post-traumatic stress disorder (PTSD). The overall research aim was to examine the relationship between these transdiagnostic factors and their relation with depression and PTSD symptoms. The systematic review was conducted in accordance with the Preferred Reporting Items for Systematic Review and Meta-Analyses (PRISMA) guidelines. Out of the 768 articles initially identified, 55 met the inclusion criteria for the current review. The results determined that intolerance of uncertainty is indirectly related to depression and PTSD symptoms, mainly through other factors including emotion dysregulation and rumination. Additionally, emotional dysregulation is a significant predictor of both depression and PTSD symptoms. Rumination is a robust factor related to depression and PTSD symptoms, this relationship was significant in cross-sectional and longitudinal studies. This review provides evidence on the transdiagnostic factors of intolerance of uncertainty, emotional dysregulation and rumination in the relationship with depression and PTSD symptoms.

## Epidemiology prevalence and comorbidity

Depressive, anxiety, and substance use disorders are among the leading contributors to the global disease burden (Whiteford et al., 2013). Depression is one of the most common mental disorders, with an estimated prevalence of 4.4% for the past year (WHO, 2017). Mental disorders rarely occur in isolation, particularly major depression disorder (MDD) has shown elevated comorbidity rates among different psychopathologies (Watson, 2009). This pattern of comorbidity can be seen with post-traumatic stress disorder (PTSD). Epidemiological studies indicate that around 80% of patients with PTSD may have comorbid disorders including depression, anxiety, or substance abuse (Foa et al., 2000). Among individuals who meet the criteria for PTSD diagnosis 48% to 55% also meet the criteria for MDD (Elhai et al., 2008; Kessler et al., 1995). This elevated comorbidity rate persisted even after controlling for overlapping symptoms (Brady et al., 2000; Elhai et al., 2008; Grubaugh et al., 2010).

Individuals with comorbid symptoms of depression and PTSD, when compared to individuals with only one type of symptom group, report lower levels of functioning, more severe symptoms, and poorer treatment outcomes (Brady et al., 2000). The presence of comorbidity entails difficulties for their diagnosis, clinical management, greater probabilities of resistance to treatment, recurrence, and use of health resources (Clark et al., 2012). However, despite the evidence of elevated comorbidity rates and considerable amount of symptom overlaps between depression and PTSD (Brady et al., 2000), the study of vulnerability factors has generally focused on their individual contribution to psychopathology (Hong & Cheung, 2015). However, less is known about basic psychological processes that may contribute to this comorbidity. Thus, it is important to understand what psychological factors contribute to the co-occurrence of depression and PTSD.

## Transdiagnostic factors

Transdiagnostic models of psychopathology emerged in response to the evidence of the lack of specificity of vulnerability factors to explain individual mental disorders, as well as the overlap of symptoms of presumably categorically different disorders. A transdiagnostic approach to psychopathology aims to understand different mental disorders or consistent groups of mental disorders based on similar cognitive, behavioral, and physiological processes involved in their etiology (Sandín et al., 2012). Moreover, this approach has allowed generating a framework with a flexible conceptualization that facilitates the understanding of the comorbidity patterns present in psychopathology, based on common variables associated with the development, maintenance, and treatment (Aldao, 2012). The study of common vulnerability factors among disorders will make it possible to understand comorbidity, better define current diagnostic classification systems, and design appropriate treatment strategies for a wide range of disorders. Several vulnerability factors have shown associations with multiple psychopathological symptoms. Particularly, for symptoms of depression and PTSD, various factors of a transdiagnostic nature have been identified including: intolerance of uncertainty (Carleton et al., 2012; Gentes & Ruscio, 2011), emotional dysregulation (Sloan et al., 2017), and rumination (Aldao et al., 2010; Olatunji et al., 2013).

## Intolerance of uncertainty

Intolerance to uncertainty is the dispositional inability of an individual to withstand the response triggered by the perceived absence of relevant, key or sufficient information (Carleton, 2016). Individuals with high levels of intolerance of uncertainty are more likely to interpret uncertainty negatively (Carleton et al., 2007). Moreover, uncertainty may contribute to maladaptive emotional, cognitive and behavioral processes that are associated with emotional distress, such as general anxiety disorder and panic disorder, (Boswell et al., 2013; Buhr & Dugas, 2009). Intolerance of uncertainty is a two-dimensional construct consisting of the prospective and the inhibitory dimensions. The prospective dimension has an anticipatory cognitive nature and is conceptualized as a desire for predictability of future events. The inhibitory dimension refers to behavioral paralysis and impaired functioning due to uncertainty (Carleton et al., 2007). Evidence from multiple studies indicating substantial associations between intolerance of uncertainty and internalizing disorders, has prompted researchers to conceptualize intolerance of uncertainty as a major transdiagnostic risk factor in these conditions (Shapiro et al., 2020). A recent meta-analysis underscored the transdiagnostic nature of intolerance of uncertainty, finding strong and significant associations between intolerance of uncertainty and symptoms of depression, anxiety, and eating disorders (McEvoy et al., 2019). Intolerance of uncertainty has also been suggested as a vulnerability risk factor for elevated PTSD symptoms (Banducci et al., 2016; Oglesby et al., 2016).

## Emotion dysregulation

Emotional dysregulation is a multidimensional construct that involves maladaptive ways of responding to emotions, including (a) lack of awareness, understanding, and acceptance of emotions; (b) the inability to control behaviors when experiencing emotional distress; (c) lack of access to situationally appropriate strategies to modulate the duration and/or intensity of emotional responses to meet individual goals and situational demands; and (d) unwillingness or reluctance to experience emotional distress as part of seeking meaningful activities in life (Gratz & Roemer, 2004). People who have difficulties regulating their emotions are more likely to engage in maladaptive behaviors such as impulsivity, avoidance, substance use, and other risky behaviors (Weiss et al., 2012). Difficulties with emotional regulation have also demonstrated their transdiagnosis role in a range of psychopathologies including depression (Aldao & Nolen-Hoeksema, 2010), substance use disorder (Wong et al., 2013) and PTSD (Seligowski et al., 2014). Specifically, Aldao and Nolen-Hoeksema (2010) examined the specificity of cognitive emotional regulation strategies from a transdiagnostic perspective. They found that dysfunctional emotional regulation strategies were associated with indicators of psychopathology symptoms (eating, depressive and anxious), this association remained regardless of the specific disorder.

## Rumination

Rumination is defined as repetitive and passive thoughts about negative emotions, their possible consequences and causes (Nolen-Hoeksema, 2000; Nolen-Hoeksema et al., 2008). Rumination prevents active problem solving to change the situations causing these negative emotions. Consequently, individuals focus on the problems and their feeling about them rather than taking action (Nolen-Hoeksema et al., 2008). It has been suggested that rumination consists of two components: reflection, which indicates a tendency to focus on one’s thoughts in order to find a solution to the problem or situation that distresses the individual, and brooding, which refers to a passive reflection were no action is taken to improve the situation (Treynor et al., 2003). Rumination has been traditionally associated with depressive symptoms and has been considered an important factor in the onset, maintenance, and recurrence of depression (Nolen-Hoeksema et al., 2008; Olatunji et al., 2013). Individuals who engage in ruminative processes report greater levels of depressive symptoms over time (Nolen-Hoeksema, 2000). Rumination has also been identified as a maladaptive cognitive style associated with multiple disorders and symptoms (Aldao et al., 2010). Particularly, for PTSD symptoms it is considered an important factor that maintains and increases symptom severity (Szabo et al., 2017).

## The present review

As previously mentioned, depression and PTSD are highly comorbid disorders with more than half of the people with a lifetime diagnosis of PTSD also meeting the criteria for a diagnosis of MDD (Elhai et al., 2008). This elevated comorbidity rate persisted even after controlling for overlapping symptoms (Brady et al., 2000; Elhai et al., 2008; Grubaugh et al., 2010). Transdiagnostic models and the study of vulnerabilities factors between depression and PTSD symptoms is important for better understanding of comorbidity, improving theoretical models of comorbidity and guiding clinical intervention. Thus, it is crucial to investigate how these factors play a role in the relationship between these highly comorbid disorders. The current systematic review sought to identify quantitative empirical studies that focused on the transdiagnostic factors of intolerance of uncertainty, emotional dysregulation and rumination, and their relation with symptoms of depression and/or PTSD. The overall research aim was to examine the relationship between these transdiagnostic factors and depression and /or PTSD symptoms.

## Method

### Literature search

The review was conducted in accordance with the Preferred Reporting Items for Systematic Review and Meta-Analyses (PRISMA) guidelines (Liberati et al., 2009). Studies were identified by searching multiple databases including PsychInfo, Medline, Web of Science, and Google Scholar during June and July 2021. Keywords/Search terms included various combinations of ‘intolerance of uncertainty’, ‘emotion regulation’, ‘emotional dysregulation’, ‘rumination’, ‘depression’, and ‘posttraumatic stress disorder’.

#### Inclusion and exclusion criteria

**Fig. 1:**
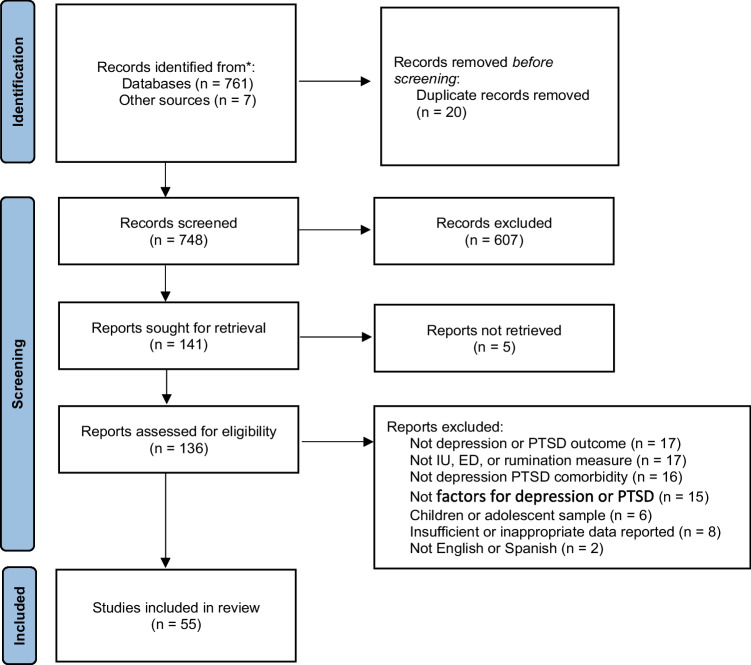
PRISMA diagram of articles selected for review. *Note:* IU = intolerance of uncertainty; ED = emotional dysregulation; PTSD = Post-Traumatic Stress Disorder

Eligible empirical studies were included if they met the following inclusion criteria: (1) Empirical quantitative research reports published in a peer reviewed journal (2) Written in English or Spanish language, (3) Outcome measures related to depression or PTSD symptoms or diagnosis. Studies were excluded if the following criteria was met: (1) No depression or PTSD outcome measure, (2) No rumination, intolerance of uncertainty or emotional dysregulation measure, (3) Not depression PTSD comorbidity, (4) Children or adolescent sample, (5) Insufficient or inappropriate data reported (Fig. 1, Table 1).

**Table 1:** Studies included in the systematic review

Study	N	Sample characteristics	Design	Transdiagnostic variable measure	Outcome measure	Key findings
Paulus et al. (2015)	642	Community	Cross-sectional	IU (IUS)	Depression (BDI-II)	IU mediates the relationship between negative affect among several emotional disorders including depression
McEvoy and Erceg-Hurn (2016)	108	Community	Experimental	IU (IUS-12)	Depression (BDI-II)	Changes in IU were associated with reductions in repetitive negative thinking, but not with depressive symptoms
Dar et al. (2017)	120	Clinical	Cross-sectional	IU (IUS)	Depression (BDI-II)	Worry mediated and moderated the relationship between IU and symptoms of depression
Swee et al. (2018)	221	Undergraduate	Cross-sectional	IU (IUS-12)	Depression (BDI-II)	IU is indirectly associated to depressive symptoms through worry and trait anxiety
Toro et al. (2018)	506	Community	Cross-sectional	IU (IUS)	Depression (BDI-II)	Negative affect (state and trait) is a partial mediator of the relationship between II and depressive symptoms
Barry et al. (2019)	66 48	Community Clinical	Cross-sectional	IU (IUS-12)	Depression (BDI-II)	IU and constructive and unconstructive rumination are predictors of depressive symptoms even when anxiety symptoms were accounted for. However, once anxiety symptoms were accounted for, they did not contribute to a depression diagnosis
Huang et al. (2019)	494 321	Community	Study 1 Cross-sectional Study 2 Longitudinal	IU (IUS-12)	Depression (PHQ-9)	Rumination partially mediated the relationship between IU and depressive symptoms. However, rumination fully mediated this relationship over two months
Saulnier et al. (2019)	374	Clinical	Cross-sectional	IU (IUS-12)	Depression (BDI-II)	IU general factor related with cognitive and affective/somatic depressive symptoms. While the inhibitory dimension of IU only related to the cognitive depressive symptoms
Del Valle et al. (2020)	3805	Community	Cross-sectional	IU (IUS)	Depression (BDI-II)	IU was a significant predictor of depressive and anxiety symptoms in the context of COVID-19 pandemic
Voitsidis et al. (2021)	2827	Community	Cross-sectional	IU (IUS)	Depression (PHQ-9)	Fear of COVID-19 partially mediated the association between IU and depressive symptoms
Chen et al. (2021)	56 53	Community Clinical	Cross-sectional	IU (IUS-12)	Depression (HAMD)	Maladaptive negative metacognitive beliefs mediate the effect of IU on depression symptoms
Oglesby et al. (2016)	50	Undergraduate	Longitudinal	IU (IUS)	PTSD (PCL-C)	Pre-trauma IU is a significant predictor of elevated post-trauma PTS symptoms following a campus shooting, even after covarying for pre-trauma levels of anxiety sensitivity
Boelen et al. (2016)	134	Community	Longitudinal	IU (IUS-12)	PTSD (PSS-SR)	Inhibitory IU positively related to levels of PTS and depression symptoms, even when controlling for neuroticism, worry, and rumination. Prospective IU predicted prolonged grief disorder severity six month later but not PTSD or depression
Oglesby et al. (2017)	126	Community	Cross-sectional	IU (IUS-12)	PTSD (PCL-C)	IU was associated with an increase in PTSD symptoms (except for re-experiencing), even after covarying for negative affect and anxiety sensitivity
Boelen (2019)	193	Undergraduate	Longitudinal	IU (IUS-12)	PTSD (PSS-SR)	Inhibitory IU pre-event predicted post-event PTSD symptoms (except for the PTSD re-experiencing dimension)
Raudales et al. (2020)	259	Trauma exposed	Longitudinal	IU (IUS)	PTSD (PCL-C)	Anxiety sensitivity, but not distress tolerance and intolerance of uncertainty, was a significant mediator between emotion dysregulation and 1‐month follow‐up PTSS
Badawi et al. (2021)	123	Clinical	Experimental	IU (IUS-12)	PTSD (PCL-5)	Decreased in IU and inhibitory IU were associated with decreases in PTSD severity. However, prospective IU only associated with changes in re-experiencing, avoidance, and arousal PTSD symptom clusters
Abravanel and Sinha (2015)	745	Community	Cross-sectional	ED (DERS)	Depression (CES-D)	Emotion dysregulation mediated the relationship between cumulative adversity and depressive symptoms independent of risk status
Ouimet et al. (2016)	150	Undergraduate	Cross-sectional	ED (DERS)	Depression (DASS)	Emotion dysregulation and maladaptive belief about emotions mediated the relationship between anxiety sensitivity and depressive symptoms
Pickard et al. (2016)	151	Undergraduate	Cross-sectional	ED (DERS)	Depression (DASS)	Mindfulness and emotional regulation fully mediated the relationship between three attachment styles (secure, preoccupied and dismissive) and depressive symptoms. However, fearful attachment partially mediated this relationship
Diedrich et al. (2017)	69	Clinical	Cross-sectional	ER (ERSQ)	Depression (BDI-II)	The ability to tolerate negative emotions was the only emotional regulation skill that mediated the relationship between self-compassion and depressive symptoms
Mutz et al. (2017)	364	Community	Cross-sectional	ER (ERQ)	Depression (PHQ-9)	Expressive suppression mediated the relationship between mental toughness and depressive symptoms
Khakpoor et al. (2019)	26	Clinical	Experimental	ER (DERS)	Depression (BDI-II)	The Unified Protocol reduced depression in patients through improvement in emotion regulation. Difficulty engaging in goal-directed behavior and lack of emotional clarity, predicted 72% of variance in depression scores
Diehl et al. (2020)	911	Community	Cross-sectional	ED (DERS)	Depression (CUDOS)	The relationship between emotion dysregulation and depression symptoms remained significant, when controlling for baseline mindfulness. However, when controlling for baseline emotion dysregulation, the association between mindfulness and depression was not significant in the majority of cases
Groarke et al. (2021)	522	Community	Longitudinal	ED (DERS)	Depression (PHQ-9)	In the context of COVID-19 loneliness predicted higher depressive symptoms one month later, and depressive symptoms predicted higher loneliness one month later. This relationship was not mediated by emotion regulation difficulties. However, emotion regulation difficulties and depressive symptoms were reciprocally related
O’Bryan et al. (2015)	297	Undergraduate	Cross-sectional	ED (DERS)	PTSD (PDS)	Difficulties with emotional acceptance significantly predicted greater avoidance and hyperarousal symptom severity above and beyond the effects of number of trauma types and negative affect. Emotion dysregulation was not significantly predictive of reexperiencing symptom severity
Short et al. (2016)	746	Trauma exposed	Cross-sectional	ED (DERS)	PTSD (PDS)	Impulse control difficulties were associated across PTSS clusters (re-experiencing, avoidance, and hyperarousal), while lack of emotion regulation strategies and emotional clarity were uniquely associated with numbing symptoms, after covarying for neuroticism
Raudales et al. (2019)	209	Community	Cross-sectional	ED (DERS)	PTSD (PCL-C)	Emotion dysregulation mediates the effects of trauma type on PTSD symptoms for sexual assault but no other trauma types, this effect remained significant after covarying for negative affect
Forbes et al. (2020)	85	Trauma exposed	Longitudinal	ER (DERS)	PTSD (PCL-5)	Emotion dysregulation predicted PTSD symptom severity at 3 months, even after covarying other risk factors (age, gender, race, ethnicity, trauma type, childhood adversity or trauma exposure, and lifetime trauma exposure) and baseline PTSD symptoms
Pencea et al. (2020)	135	Trauma exposed	Longitudinal	ER (EDS-short)	PTSD (PSS)	Emotion dysregulation predicted chronic PTSD symptom, even after controlling for trauma exposure, baseline PTSD and depressive symptoms
Fujisato et al. (2020)	1794	Community	Longitudinal	ER (ERSQ)	PTSD (PCL-5)	Emotion regulation predicted PTSS 4-months later, even after controlling for symptoms at baseline
Post et al. (2021)	200	Clinical	Cross-sectional	ER (ERQ)	PTSD (PSS-I)	Emotion regulation fully mediated the relationships between negative affect and PTSD and MDD, and negative mood regulation expectancies and PTSD and MDD
Iqbal and Dar (2015)	77	Clinical	Cross-sectional	Rumination (RRS)	Depression (BDI)	Brooding and reflection rumination mediated the association between negative affect and depressive symptoms, but not anxiety
Vanderhasselt et al. (2016)	92	Undergraduate	Longitudinal	Rumination (RRS)	Depression (BDI-II)	Co-variation of stressful events and rumination predicted depressive symptoms at 3 and 15 months. This effect remained even when statistically controlling for baseline depressive symptoms
Petrocchi and Ottaviani (2016)	41	Undergraduate	Longitudinal	Rumination (RRS)	Depression (CES-D)	Rumination was a significant mediator of the relationship between nonjudge (mindfulness facet) and depressive symptoms after two years
Liu et al. (2017)	87	Clinical	Cross-sectional	Rumination (RRS)	Depression (HAMD)	Rumination partially mediated the relationship between overgeneral autobiographical memory and depressive symptoms. Particularly, maladaptive brooding subtype of rumination
Vine and Marroquin (2017)	100	Clinical	Cross-sectional	Rumination (RRS)	Depression (MASQ AD)	Rumination mediated associations of emotional clarity with depressive symptoms regardless of affect intensity
Schut and Boelen (2017)	208	Undergraduate	Longitudinal	Rumination (RRS)	Depression (BDI-II)	Trait mindfulness, but not brooding, reflection, and experiential avoidance predicted depressive symptoms after one year, while controlling for baseline depression symptoms
Senra et al. (2017)	438	Community	Cross-sectional	Rumination (RRS)	Depression (BDI-II)	Brooding-rumination and immature defenses mediated the relationship between perfectionism and depressive symptoms. Furthermore, brooding-rumination moderated the impact of perfectionism on depressive symptoms
Costa et al. (2018)	70 70	Clinical Community	Cross-sectional	Rumination (RRS)	Depression (DASS)	Cognitive fusion, but not rumination and mindfulness, was the only significant mediator of the relationship between negative affect and depressive symptoms
Bakker et al. (2018)	100	Clinical	Cross-sectional	Rumination (RRS)	Depression (PHQ-9)	Brooding rumination, experiential avoidance, and acceptance mediated the relationship between self-compassion and depressive symptoms
Whisman et al. (2020)	5891	Community	Longitudinal	Rumination (RRS)	Depression (PHQ-9)	Rumination predicted residual change in depressive symptoms and depressive symptoms predicted residual change in rumination (4-year follow-up), suggesting that rumination and depressive symptoms influence one another in a bidirectionally
Liang et al. (2020)	501	Undergraduate	Cross-sectional	Rumination (RRS)	Depression (CES-D)	Peace of mind and rumination fully-mediated the relationship between gratitude and depression, this mediation model did not differ by gender
Lyon et al. (2020)	3043	Community	Cross-sectional	Rumination (RRS)	Depression (BSI)	Brooding mediated the effect of neuroticism, extroversion, conscientiousness and openness on depressive symptoms. Reflection mediated the effects of neuroticism, extroversion and openness on depressive symptoms
De Rosa et al. (2021)	151 42	Undergraduate Clinical	Cross-sectional	Rumination (RRQ)	Depression (BDI-II)	Perfectionism is associated with rumination, in both the clinical and nonclinical populations. Rumination mediated the relationship between maladaptive perfectionism and depression
Spinhoven et al. (2015)	359	Trauma exposed	Longitudinal	Rumination (RUM)	PTSD (PSS-I)	Pre-trauma depression severity and trait rumination (but not trait worry) predicted onset of PTSD during four-year follow-up. Cognitive appraisal of the traumatic event partially mediated the association between trait rumination and PTSD
Wu et al. (2015)	318	Trauma exposed	Cross-sectional	Rumination (RRS)	PTSD (M-PTSD)	Brooding rumination and depressed-related rumination are related with higher level of PTSD
Basharpoor et al. (2015)	99	Trauma exposed	Cross-sectional	Rumination (RRS)	PTSD (M-PTSD)	Experimental avoidance and rumination in the group with PTSD were higher than those without PTSD. Mindfulness was significantly lower in the group with PTSD than without PTSD
Roley et al. (2015)	45	Trauma exposed	Cross-sectional	Rumination (RTSQ)	PTSD (PCL-5)	Repetitive rumination and anticipatory rumination moderates the relationship between PTSD and MDD symptoms
Seligowski et al. (2016)	403	Community	Cross-sectional	Rumination (RRS)	PTSD (PCL-5)	Rumination was significantly related to each PTSD symptom clusters, even after controlling for negative affect
Viana et al. (2017)	182	Trauma exposed	Cross-sectional	Rumination (RRS)	PTSD (PDS)	Mindful attention was a significant moderator of relations between rumination and all subfactors of PTSD symptoms (re-experiencing, avoidance, arousal, and total PTSD symptoms)
García et al. (2018)	629	Community	Cross-sectional	Rumination (RRS)	PTSD (SPRINT-E)	Intrusive rumination mediated the relationship between negative rumination and posttraumatic stress symptoms
Pugach et al. (2019)	90	Community	Cross-sectional	Rumination (RRS)	PTSD (CAPS-5)	Rumination fully mediated the relationship between overall emotional dysregulation and PTSD severity
Mathes et al. (2020)	119	Trauma exposed	Longitudinal	Rumination (RRS)	PTSD (PCL-C)	Hostility temporally mediated the prospective association between rumination and PTSD symptoms, even when controlling depressive disorder diagnosis
Preston et al. (2021)	204	Trauma exposed	Longitudinal	Rumination (RQ)	PTSD (PDS)	Interpersonal trauma moderated the relationship between baseline rumination and 1-month trauma symptoms, even after covarying for age and sex, treatment condition, negative affect, and number of previously experienced traumas

*IU* Intolerance of uncertainty; *ED* Emotional dysregulation; *ER* Emotional; *PTSD* Posttraumatic stress disorder; *IUS* Intolerance of uncertainty scale; *IUS-12* Intolerance of uncertainty scale short version; *DERS* Difficulties in emotion regulation scale; *EDS-Short* Emotion dysregulation scale, short version; *ERSQ* Emotion regulation skills questionnaire; *BDI-II* Beck depression inventory-II; *PHQ-9* Patient health questionnaire-9; *HAMD* Hamilton depression rating scale; *BSI* Brief symptom inventory; *CES-D* Center for epidemiologic studies depression scale; *DASS* Depression anxiety stress scales, *CUDOS* Clinically useful depression outcomes scale; *MASQ AD* Anhedonic depression subscale of the mood and anxiety symptom questionnaire short form; *PCL-5* Posttraumatic stress disorder checklist for DSM-5; *PCL-C* Posttraumatic stress disorder checklist; *M-PTSD* Mississippi post-traumatic stress disorder scale; *PSS-I* PTSD symptom scale—interview version; *CAPS-5* Clinician‐administered PTSD scale‐5; *PSS* Posttraumatic stress disorder symptom scale; *PSS-SR* PTSD symptom scale–self-report version; *PDS* Posttraumatic diagnostic scale; *SPRINT-E* Short *posttraumatic* stress disorder rating interview; *RRS* Ruminative response scale; *RUM* Subscale rumination on sadness of the revised version of the Leiden index of depression sensitivity; *RTSQ* Ruminative thought style questionnaire; *RQ* Rumination questionnaire; *RRQ* Rumination reflection questionnaire

## Results

### Study characteristics

The majority of studies were conducted in North America (43.6%), specifically USA (21 studies), and Canada (3 studies). Followed by 25.5% from European countries, 16.4% from Asia including China (4 studies), Japan (1 study), India and Iran (2 studies each), 7.2% from Oceania particularly, Australia (4 studies), and 7.2% from South America including Argentina (2 studies), Colombia and Chile (1 study each). Community-based studies were the most frequent type (39%), followed by undergraduate student sample (27.1%), clinical sample (18.6%), and trauma exposed sample (15.3%). Most of the studies were cross-sectional (66.1%), followed by longitudinal (28.6%) and a minority were experimental (5.4%).

### Intolerance of uncertainty and depression

Eleven studies examined the relation between intolerance of uncertainty and depression symptomatology. Empirical evidence indicates that intolerance of uncertainty is a significant predictor of depression symptoms, even when anxiety symptoms where accounted for (Barry et al., 2019; Del Valle et al., 2020). Furthermore, the relationship between intolerance of uncertainty and depression was indirect and partially mediated by other risk factors, including worry (Dar et al., 2017; Swee et al., 2018), trait anxiety (Swee et al., 2018), rumination (Huang et al., 2019), and fear of COVID-19 (Voitsidis et al., 2021). Two risk factors suggested a complete mediation of the effects of intolerance of uncertainty on depression symptoms: maladaptive metacognitive beliefs (Chen et al., 2021) and rumination over two months (Huang et al., 2019). Lower-order dimension analysis of intolerances of uncertainty and a two-factor model of depression indicated that a general factor of intolerance of uncertainty was related to cognitive and affective/somatic factors of depression symptoms (Saulnier et al., 2019). However, the Inhibitory dimension of intolerance of uncertainty was only related to the cognitive factor of depression, while the Prospective dimension was not related to any of these depression factors. Worry was the only risk factor that moderated the relationship between intolerance of uncertainty and symptoms of depression, consequently high levels of worry heightened the association between intolerance of uncertainty and symptoms of depression (Dar et al., 2017). An experimental treatment study determined that reductions in intolerance of uncertainty were associated with reductions in repetitive negative thinking, but not with depression symptoms (McEvoy & Erceg-Hurn, 2016).

### Intolerance of uncertainty and post-traumatic stress

Six studies that examined the relation between intolerance of uncertainty and PTSD symptoms. This evidence indicates that intolerance of uncertainty is a significant predictor of PTSD symptoms. These result remained significant even after covarying for other PTSD risk factors including: rumination, neuroticism, and worry (Boelen et al., 2016), anxiety sensitivity (Oglesby et al., 2016, 2017) and negative affect (Oglesby et al., 2017).

When the relation between intolerance of uncertainty and PTSD symptoms DSM-V clusters was examined, there was a significant association to the avoidance, hyperarousal and emotional numbing symptom clusters (Oglesby et al., 2017). Dimension analysis of intolerances of uncertainty indicated that only the inhibitory dimension was associated with PTSD symptoms (Boelen, 2019; Boelen et al., 2016). Further, a decrease in overall and inhibitory dimension was associated with decreases in PTSD severity and at the symptom cluster level. However, the prospective dimension only associated with changes in the re-experiencing, avoidance, and hyperarousal clusters (Badawi et al., 2021). Finally, mediation analysis determined that intolerance of uncertainty was not a significant mediator between distress tolerance and PTSD symptoms (Raudales et al., 2020).

### Emotion dysregulation and depression

We identified 8 studies that assessed the relation between emotion dysregulation and depression symptoms. Results indicated that emotion dysregulation was positively associated with depressive symptoms, even when controlling for baseline mindfulness (Diehl et al., 2020). Furthermore, emotional dysregulation partially and significantly mediated the relationship between depression and various factors related to depressive symptomatology, including, cumulative adversity (Abravanel & Sinha, 2015), anxiety sensitivity (Ouimet et al., 2016), maladaptive beliefs about emotions (Ouimet et al., 2016), and attachment style (Pickard et al., 2016). One study examining which emotion regulation skill mediated the association between self-compassion and depression, determined that only the ability to tolerate negative emotions was a significant mediator (Diedrich et al., 2017). Likewise, expressive suppression was the only emotion regulation strategy that mediated the relationship between mental toughness and depressive symptoms (Mutz et al., 2017). Khakpoor et al. (2019) conducted an experimental study that examined how the Unified Protocol for transdiagnostic treatment of emotional disorders reduced depression in patients through improvement in emotion regulation. Emotion regulation, particularly difficulty engaging in goal-directed behavior and lack of emotional clarity, predicted most of the variance in depression scores. Furthermore, mediation analysis concluded that emotion regulation can be considered a mediating factor and a predictive of outcomes of transdiagnostic treatment based on the Unified Protocol (Khakpoor et al., 2019). Finally, in the context of COVID-19, loneliness predicted higher depressive symptoms and depressive symptoms predicted higher loneliness one month later. This relationship was not mediated by emotion regulation difficulties. Instead, emotion regulation difficulties and depressive symptoms were reciprocally related (Groarke et al., 2021).

### Emotion dysregulation and post-traumatic stress

Seven studies assessed the relation between emotional dysregulation and PTSD symptoms. Longitudinal assessments found that emotional dysregulation was significantly associated with the probability of developing PTSD symptoms 4 months later (Fujisato et al., 2020) and at 12 months (Pencea et al., 2020), even when controlling for baseline PTSD, trauma exposure, and depressive symptoms. Likewise, emotion dysregulation predicted PTSD symptom severity at 3 months following exposure to a traumatic event, even after covarying for baseline PTSD symptoms and other risk factors such as, trauma type, childhood adversity, trauma exposure, lifetime trauma exposure, age, gender, race, and ethnicity (Forbes et al., 2020). Mediation analysis determined that emotional regulation mediated the effects of trauma type, particularly sexual assault, and PTSD symptoms (Raudales et al., 2019) and negative affect and PTSD and MDD (Post et al., 2021). When the relation between emotion dysregulation and PTSD symptom clusters were examined, emotional dysregulation only predicted avoidance and hyperarousal symptom severity even after covarying the number of trauma types and negative affect (O’Bryan et al., 2015). Difficulties in emotion regulation subscales analyses determined that difficulties in impulse control predicted re-experiencing, avoidance, and hyperarousal, whereas lack of emotion regulation strategies and emotional clarity were uniquely associated with emotional numbing symptoms (Short et al., 2016).

### Rumination and depression

Thirteen studies examined the relation between rumination and depression symptoms. Results from longitudinal associations indicated that rumination predicts depressive symptoms and that depressive symptoms predict rumination at 4-year follow-up (Whisman et al., 2020). Likewise, a correlation of stressful events and rumination predicts depressive symptoms prospectively (3 and 15 months follow-up), even when controlling for baseline depressive symptoms (Vanderhasselt et al., 2016). However, one longitudinal study found that rumination (brooding and reflection subscale) did not predict depressive symptoms one year later, when compared to trait mindfulness and while controlling for baseline depression symptoms (Schut & Boelen, 2017). Mediation analysis determined that rumination was a significant mediator between maladaptive perfectionisms (De Rosa et al., 2021), gratitude (Liang et al., 2020), non-judge mindfulness component (two years after) (Petrocchi & Ottaviani, 2016), emotional clarity (Vine & Marroquin, 2017), overgeneral autobiographical memory (Liu et al., 2017), and negative affect (Iqbal & Dar, 2015) and depression symptoms. However, rumination was not a significant mediator of the relationship between negative affect and depression, when taking into account cognitive fusion in the mediation model (Costa et al., 2018), contradicting previous research (Iqbal & Dar, 2015). Subscale analysis of rumination indicated that brooding mediated the relationship between overgeneral autobiographical memory (Liu et al., 2017), perfectionism (Senra et al., 2017), self-compassion (Bakker et al., 2018) and depression symptoms. Further, brooding mediated the effect of various personality traits such as: neuroticism, extroversion, conscientiousness and openness on depressive symptoms, while reflection mediated the effects of neuroticism, extroversion and openness on depressive symptoms (Lyon et al., 2020).

### Rumination and post-traumatic stress

Ten studies examined the relation between rumination and PTSD. Repetitive and anticipatory rumination moderated the relationship between PTSD and MDD symptoms (Roley et al., 2015). While, mindful attention moderated the relations between rumination and PTSD symptoms total, as well as all its symptom clusters (Viana et al., 2017). Interpersonal trauma moderated the relationship between baseline rumination and 1-month follow-up PTSD symptoms, even after covarying for negative affect, and number of previously experienced traumas (Preston et al., 2021). Longitudinal association determined pre-trauma depression severity and trait rumination predicted the onset of PTSD during a 4-year follow-up (Spinhoven et al., 2015). PTSD symptom clusters analysis indicated that rumination was significantly related to all symptom cluster (Seligowski et al., 2016). Mediation analysis determined that that hostility (Mathes et al., 2020), cognitive appraisal of the traumatic event (Spinhoven et al., 2015) and deliberate rumination (García et al., 2018) mediated the relationship between rumination and PTSD symptoms. Furthermore, rumination also acted as a mediator between emotional dysregulation and PTSD symptom severity (Pugach et al., 2019). Finally, experimental avoidance and rumination were compared in groups with and without PTSD, in the group with PTSD rumination was significantly higher than those without PTSD (Basharpoor et al., 2015). Brooding rumination and depressed-related rumination were related with higher level of PTSD (Wu et al., 2015).

## Discussion

The current systematic review sought to identify quantitative empirical studies that focused on the transdiagnostic factors of intolerance of uncertainty, emotional dysregulation and rumination, and their relation with depression and/or PTSD. This review identified 55 studies that reported the association between the transdiagnostic factors of interest in depression and PTSD symptoms.

Intolerance of uncertainty was a consistent significant predictor for both depression and PTSD symptoms, as suggested by other authors (McEvoy et al., 2019; Shapiro et al., 2020). This association persisted after controlling other well-known risk factors (e.g., negative affect, worry, neuroticism, and anxiety sensitivity), however anxiety sensitivity was the only variable that covaried in both depression and PTSD symptoms. This may be explained by the evidence that suggests anxiety sensitivity is broadly related to a range of internalizing disorders particularly distress disorders such as depression, GAD and PTSD (Naragon-Gainey, 2010). Mediation analysis suggested that intolerance of uncertainty and depression are indirectly related. This relationship turned out to be partially mediated by other risk factors including worry and trait anxiety. Likewise, rumination had a partial mediation role in cross sectional studies, whereas longitudinally rumination fully mediated the relation between intolerance of uncertainty and depression. Therefore, individuals with high levels of intolerance of uncertainty ruminate over time as an attempt to cope with negative emotions, meanwhile increasing the risk of developing depressive symptoms (Huang et al., 2019). Only one study conducted a mediation analysis taking into consideration intolerance of uncertainty as a mediator between emotional dysregulation and PTSD symptoms, however this relation was not significant. This result suggests that intolerance of uncertainty may be indirectly related to PTSD symptoms possibly through other risk factors, in a similar way as found in depression symptoms. Lower-order dimension analysis of intolerances of uncertainty indicated that the inhibitor dimension was only related to cognitive factors of depression and PTSD clusters avoidance and hyperarousal. This result supports the notion that the inhibitory dimension refers to impaired functioning due to uncertainty. While the prospective dimension was not related to any individual factors of depression, it was related to avoidance, hyperarousal and re-experiencing PTSD symptoms. Particularly, this dimension has an anticipatory cognitive nature and is conceptualized as a desire for predictability of future events.

Emotional dysregulation has been found to relate to depression as well as many other psychiatric symptoms including PTSD (Aldao et al., 2010). In this systematic review emotional dysregulation was a significant predictor of both depression and PTSD symptoms. For depression symptoms this result was significant also after covarying for mindfulness. In relation to PTSD symptoms, longitudinal analyses determined that emotional dysregulation remained a significant predictor also after covarying other well established PTSD risk factors including, baseline PTSD symptoms, depressive symptoms, trauma type, trauma exposure, childhood adversity, lifetime trauma exposure, age, gender, race, and ethnicity. Furthermore, emotional dysregulation has a mediating effect between several risk factors and depression or PTSD symptoms such as, cumulative adversity, anxiety sensitivity, attachment style, maladaptive beliefs about emotions, sexual assault and negative affect. PTSD symptom cluster analysis indicated that emotional dysregulation predicted avoidance and hyperarousal symptoms but not re-experiencing, even when covarying for number of trauma types endorsed and negative affect. Specific associations between facets of difficulties in emotion regulation and PTSD symptom clusters determined that difficulties controlling impulses while distressed were associated with reexperiencing, avoidance, and hyperarousal symptoms. Therefore, individuals with PTSD symptoms may engage in maladaptive behaviors to help regulate negative emotions such as impulsivity, avoidance, substance use and other risky behaviors (Weiss et al., 2012). Moreover, lack of effective emotion regulation strategies and lack of emotional clarity were related only to emotional numbing PTSD symptoms. This result is also consistent with findings that emotional regulation strategies are related to depression symptoms such as, loss of interest, feeling emotionally numb, and the inability of experiencing positive emotions (Aldao et al., 2010).

Rumination is an important factor related to the maintenance and exacerbation of depression and PTSD symptoms (Olatunji et al., 2013; Szabo et al., 2017). Longitudinal studies determined that rumination predicted both depression and PTSD symptoms at 4-year follow-up, even after accounting for the effects of baseline depressive symptoms. It should be noted that depression symptoms and rumination influenced one another bidirectionally, that is rumination predicts depressive symptoms and depressive symptoms predict rumination. Rumination has been found to mediate the relationship between depression and several factors related to depression symptoms including, maladaptive perfectionism, gratitude, overgeneral autobiographical memory, mindfulness, emotional clarity, and negative affect. These findings support the theoretic notion that rumination maintains distress, especially depression, through a variety of mechanisms (Nolen-Hoeksema et al., 2008). Evidence for PTSD symptoms indicated that rumination only acted as a mediator between emotional dysregulation and PTSD symptom severity. However, the relationship between rumination and PTSD symptoms was mediated by other factors such as hostility, cognitive appraisal of the traumatic event, and deliberate rumination. PTSD symptom clusters analysis determined that rumination was significantly related to all symptom clusters, which highlights the importance of rumination in individuals who have been through a traumatic event and who may be at greater risk of developing PTSD symptoms. These studies also found that mindfulness and interpersonal trauma moderated of the relationship between rumination and PTSD symptoms. Consequently, interpersonal trauma enhances the association between rumination and PTSD symptoms, while mindfulness weakens this relation. Furthermore, rumination moderated the relationship between PTSD and MDD symptoms, this implies that higher levels of rumination in individuals with PTSD are more likely to also have greater depressive symptoms.

### Limitations

This systematic review had a number of limitations that should be taken into account when interpreting the results and addressed in future research. First, even though studies in Spanish were included in an attempt broaden the scope, the majority of the studies were conducted in North American and European countries. Furthermore, taking into consideration the evidence that culture may play a significant role in the manifestation of psychopathology (Rathod, 2017), the results of this study may have limited generalizability to other cultural contexts. Thus, there is a compelling need for future research in other regions of the world to better understand the impact of culture on psychopathology. Second, although elevated comorbidity rates between depression and PTSD symptoms are well known, only two studies were found that addressed comorbidity in these disorders. Future research should aim to investigate factors related to the comorbidity between depression and PTSD symptoms. Third, another significant limitation was that most of studies used cross-sectional samples, making it difficult to determine a causal inference. Additionally, most of the studies were from community-based samples which are more likely to present low to moderate symptom levels, thus some studies may have underrepresented symptom levels. This review used a systematic approach. Conducting a meta-analysis on the data would be an interesting future step, to get a more comprehensive understanding of the overall effect across the transdiagnostic factors. Additionally, future studies will need to employ quality assessment of eligible studies using quality assessment tools. Finally, this study relied on questionnaires to assess the variables of interest. Future research should consider incorporating additional measures, such as biomarkers or objective assessments, to provide a more comprehensive and objective understanding of the disorders being studied. Future research should consider incorporating additional measures, such as biomarkers or objective assessments. For example, recent studies have demonstrated that near-infrared spectroscopy (fNIRS) can effectively identify diminished hemodynamic response in major depressive disorder patients, thus providing an objective assessment of depression (Husain et al., 2020; Li et al., 2022). Furthermore, fNIRS has shown potential in identifying brain markers associated with putative symptoms of PTSD (Balters et al., 2021).

## Conclusion

This review provides evidence that the transdiagnostic factors of intolerance of uncertainty, emotional dysregulation and rumination are consistent significant predictors for both depression and PTSD symptoms. Particularly, intolerance of uncertainty is indirectly related to depression and PTSD symptoms through other factors including emotion dysregulation and rumination. Meanwhile, emotional dysregulation is a significant predictor of both depression and PTSD symptoms. Rumination is a robust factor related to depression and PTSD symptoms, this relationship was significant in cross-sectional and longitudinal studies. Both emotional dysregulation and rumination can mediate the relationship between several risk factors including intolerance of uncertainty and depression and PTSD symptoms. This study provides insight into understanding these factors in the onset and maintenance of depression and PTSD symptomatology. Consequently, contributing to the evidence that allows the development of theoretical and empirically supported transdiagnostic models. Which may ultimately promote the development of cost-effective treatments that targets underlaying mechanism or factors, thus promoting preventative alternatives for multiple disorders.

## Data Availability

The datasets generated during and/or analysed during the current study are available from the corresponding author on reasonable request.
